# Cell death and regeneration in Dresden—the 27th meeting of the European Cell Death Organization

**DOI:** 10.1038/s41419-019-2153-5

**Published:** 2019-12-04

**Authors:** Andreas Linkermann, Dagmar Kulms

**Affiliations:** 1 Division of Nephrology, Department of Internal Medicine III, University Hospital Carl Gustav Carus at the Technische University of Dresden, 01069 Dresden, Germany; 2 Department of Dermatology, University Hospital Carl Gustav Carus at the Technische University of Dresden, 01069 Dresden, Germany

**Keywords:** Apoptosis, Necroptosis

Following the 2018 European Cell Death Organization (ECDO) meeting in Saint Petersburg, high expectations preceeded the 2019 conference in Dresden, Germany. From the 25th to the 27th of September, more than 200 cell-death researchers from globally distributed institutions gathered in Dresden. An interesting mixture of basic scientists, clinician scientists, clinicians, postdocs, and students set the basis for vivid discussion and a lively atmosphere.

In slight contrast to previously held training courses, the conference in Dresden was opened by a session of leading young scientists in our field termed “The future of cell death science—the young investigator presentations”, chaired by Markus Rehm. Mathieu Bertrand opened the session with a presentation on the molecular mechanisms of receptor-interacting protein kinase 1 (RIPK1) regulation that defines live and death decisions upon activation of the TNF-receptor 1 (TNFR1). In particular, the presenter focused on the role of the ubiquitin-editing enzyme A20 and the different zinc finger domains therein. These domains appear to define the type of ubiquitin chains that modify RIPK1 function. Mads Daugaard introduced current knowledge about prostate cancer progression with a focus on the role of glycosaminoglycan (GSG) enzymes, in particular CHST11. Mice deficient in this enzyme were not viable and human cancer cells die by anoikis. Christina Munoz-Pinedo presented mechanisms of starvation responses and demonstrated how glucose-deprived cancer cells release IL-8. Alexei Degterev showed details of the RIPK1 kinase, pocked and pointed out the differences between mice and humans within this structure. He also introduced CLSP37, a novel RIPK2 inhibitor. Bart Tummers demonstrated detailed studies about the origin of the autoimmune lymphoproliferative syndrome (ALPS), the pathophysiology of which has been an obstacle for decades in the cell-death research field. The integrated control of apoptosis, necroptosis, and pyroptosis appears to be dysregulated in this syondrome. Stephanie Kreis presented novel insights into drug combination therapies best to be used for melanoma treatments by combining miRNome profiling followed by qCLASH analysis (cross-linking, ligation, and sequencing of hybrids), an innovative method to determine real miRNA–mRNA interactions, and explained a novel approach to overcome drug resistance in these tumors. Jose Pedro Friedmann-Angeli described a novel key inhibitor of ferroptotic cell death that appears to function alongside of the master regulator selenoprotein glutathione peroxidase 4 (GPX4).

The second session on “Cell Death and Immunity/Inflammation” was chaired by Ivano Amelio and kicked off with the keynote lecture by Scott Lowe. He presented a fascinating overview on our understanding of the tumor suppressor p53 functions in and beyond cell death in his first part of the lecture. He subsequently pointed out novel insights about the involvement of p53 in the senescent-associated secretory phenotype (SASP), which he demonstrated to be controlled by nuclear factor kappa-B (NF-κB) and bromodomain-containing protein 4 (Brd4). Peter Vandenabeele compared the immunogenicity of apoptosis, necroptosis, and ferroptosis, challenging our current concept of immunogenic cell death and/or necroinflammation. Peter Krammer presented work on the “annexin check point system”, and Leonie Hartmann elaborated on the role of interferon-induced mixed lineage kinase domain like pseudokinase (MLKL) activation in gastrointestinal infection models. Tania Watts shed light on TNF-receptor signaling in the context of adaptive antiviral immune responses, and discussed a role for that system in cancer. Mads Gyrd-Hanssen presented on the mechanisms limiting ubiquitin-mediated post-translational modification by the cylindromatosis lysine 63 deubiquitinase (CYLD). After the coffee break, Tom Vanden Berghe presented translational cell-death science by linking basic research to clinical trials to treat IL-1β and IL-18-driven sepsis. He additionally presented spectacular novel ferroptosis inhibitors that are on their way to clinical trials. Daniel Frank generated a K469R mutant of receptor-interacting protein kinase 3 (RIPK3), a polyubiquitin site close to the RHIM domain, and unexpectedly observed increased RIPK3 phosphorylation and necroptosis, downstream of RIPK1 kinase activity.

A flash-talk session of eight-time 2-min presentations followed to especially announce some of the most interesting abstracts, before Carlo Croce set out for the 2019 CDD honorary lecture. In detail, he recapitulated the scientific endeavor to isolate BCL2. He explained the effort and roadblocks that needed to be overcome before the generation of venetoclax was actually possible. The first day ended with an open bar during the first poster session.

Simone Fulda moderated the first session on the second day of the meeting: “Cell Death and Regeneration”. Kevin Ryan started a set of exciting presentations by demonstrating how cancers can be attacked when the glucose and mannose metabolism profiles are understood. Sudan He investigated the influence of necroptosis regulators on the tumor microenvironment. Elena Morgun focused her talk on the role of caspase-3 expression in a model of chronic wound healing. Jaewhan Song connected the protein Beclin-1, best known for its role in autophagy, to the necroptosis pathway and developed a model in which Beclin-1, independent of autophagy, interacts with MLKL. Edward Mocarski continued his work on herpesvirus proteins controlling necroptosis and thereby not only limiting the death of the host cell, but also the immune response against the virus. Mechanisms of mitochondrial permeability transition and connections between apoptotic and necrotic cell death controlled by Bax/Bak were discovered by Richard Kitsis, both in cell culture assays and mouse models of heart disease. Patricia Boya analyzed previously unrecognized links between autophagy and apoptosis, before Motti Gerlic shared his results on purified extracellular vesicles released from necroptotic cells. He used this material to perform a proteomic analysis of vesicles budding from dying cells. Florian Bock finished the morning session with the presentation of his results on paracrine resistance mechanisms upon cancer therapy. Participants thereafter enjoyed Poster Session 2, lunch, and vivid discussions.

The afternoon session on “Cell Death and Cancer” was chaired by Klaus-Michael Debatin and started with a talk by Gerry Melino. He elaborated on the DNA damage response that his group discovered to be regulated by ZNF281, and defined this factor as a prognostic marker for neuroblastoma. Triona Ni Chonghaile works on HDAC6 inhibitors, which she found in a small-molecule screen in apoptosis-resistant cancer cells. Claudio Mauro introduced the metabolic control of immune-mediated inflammation and its affection upon cell death. Carol Prives presented insights on the regulation of ferroptosis by Mdm2/MdmX, a collaboration with the later keynote speaker Brent Stockwell. Andreas Villunger introduced the role of caspase-2 in cancer resistance, and Lisa Bouchier-Hayes, working on alternative functions of caspase-2, discovered this protease to function as an S-phase checkpoint regulator, potentially to allow DNA repair. Caitlin Brown showed the results on prominin 2 that regulates iron export and thereby sets the threshold for ferroptosis sensitivity. Finally, the second keynote lecture by Brent Stockwell on mechanisms and therapeutic applications of ferroptosis elegantly delineated the timeline of ferroptosis research, and introduced a novel antibody for the detection of ferroptosis. The day ended with the speakers dinner.

The third day started when Chair Boris Zhivotovsky introduced Francis Chan as the first speaker in the session “Non-apoptotic Cell Death Mechanisms”. He introduced three ways of how the growing number of pathogens target RIPK3: “Tackle it! Chop it! Shred it!”. William Kaiser employed a virally inspired screen to discover the cytomegalous virus (CMV) entry receptor and a novel set of necroptosis regulators. Anne Hamacher-Brady established the role of endolysosomal interactions with Bax-mediated mitochondrial permeability transition. Benjamin Demarco clearly demonstrated a caspase-8-dependent cleavage of gasdermin D that appeared to be independent of the inflammasome. Again, coffee. Junying Yuan presented encouraging data on the benefit of necroptosis inhibition by RIPK1 inhibitors in a model of rodent stroke and neurodegeneration. Seamus Martin focused on the cell-death-independent functions of death molecules in regulating inflammation, and pointed out the importance to differentiate between cell death and cell stress functions. Feng Shao discovered a caspase cleavage motif in gasdermin molecules that is distinct from the commen tetrapeptide, and presented the structural basis for this novel mechanism. Douglas Green extended our knowledge on LC3-associated endocytosis (LANDO), a fundamentally important autophagy-independent pathway for microglial activation and neurodegeneration.

The last session called “Structural and Systems Biology in Cell Death” was chaired by the president of the ECDO, Patrizia Agostinis. Peter Czabotar introduced his work on controlled conversion of Bax and Bak into membrane-permeabilizing executioners. Marion MacFarlane dissected the molecular architecture of FADD/caspase-8 signaling complexes in high-resolution imaging and structural modeling. Nadine Pollak, also working on extrinsic apoptosis, focused on the role of Mcl-1 in controlling caspase-8 activation during cell cycle progression. Jochen Prehn provided insights on how responses to chemotherapy can be predicted from systems analysis approaches in colorectal cancer and presented mechanistic insight into TRAIL-induced entosis as an escape mechanism of cancer cells. Last coffee of the meeting. Inna Lavrik used chemical probes designed to target the death receptor network to control extrinsic apoptosis. Judy Liberman demonstrated how microbes die by microptosis following cytotoxic lymphocyte stimulation.

Finally, it was time for the 2019 ECDO honorary lecture, presented by Shigekazu Nagata. He took us on an exciting journey over several decades of cell-death research: from the discovery of phosphatidylserine as an eat-me signal to the molecular mechanisms of the phospholipid-scrambling machinery, explaining how billions of cells in each human body are dying each day. With the next few years of meeting announcement, the celebration of the poster prizes, and the conference dinner, the 27th ECDO meeting closed down. We are looking forward to the 28th ECDO conference in the United Kingdom.

